# Mechanical Properties of TC11 Titanium Alloy and Graphene Nanoplatelets/TC11 Composites Prepared by Selective Laser Melting

**DOI:** 10.3390/ijms23116134

**Published:** 2022-05-30

**Authors:** Bingxian Ou, Lixin Lu, Qinsheng Wang, Qing He, YiLin Xie, Junxia Yan

**Affiliations:** 1 School of Mechanical Engineering, Jiangnan University, Wuxi 214122, China; obxobx@163.com (B.O.); lulx@jiangnan.edu.cn (L.L.); 6200810044@stu.jangnan.edu.cn (Q.H.); 2 Special Equipment Safety Supervision Inspection Institute of Jiangsu Province, National Graphene Products Quality Inspection and Testing Center (Jiangsu), Wuxi 214174, China; wqs@wxtjy.com (Q.W.); 18018309369@189.cn (Y.X.); 3 Jiangsu Province Engineering Research Center of Micro-Nano Additive and Subtractive Manufacturing, Wuxi 214122, China; 4 Jiangsu Key Laboratory of Advanced Food Manufacturing Equipment and Technology, Wuxi 214122, China

**Keywords:** selective laser melting, mechanical property, GNPs/TC11 titanium matrix composite, graphene, microstructure

## Abstract

Titanium matrix composites (TMCs) with excellent mechanical properties, reinforced by graphene, is deemed the lightweight and high strength structural materials. In this study, TC11 titanium alloy powder and graphene nanosheets (GNPs) were used as raw materials, and the composite powder with good uniformity and fluidity was obtained through non-interventional homogeneous mixing by a planetary mixer. The microstructure and mechanical properties of the GNPs-TC11 composites and TC11 alloy were compared. The results showed that the microstructure of TC11 and the composites was acicular martensite α’ phase under the process parameters of 280 W laser power, 1200 mm/s scanning speed, and 0.1 mm hatch spacing. The GNPs in addition, in the composites, reduced the acicular martensite particle size and expanded the proportion of low-angle grain boundaries. The tensile strength and percentage elongation after the fracture of the TC11 titanium alloy were 1265 MPa and 4.3%, respectively. Because of addition of the GNPs, the strength and percentage elongation after the fracture of the composite increased to 1384 MPa and 8.1%, respectively, at a GNPs mass content of 0.2%. The enhancement of mechanical properties can be attributed to grain refinement, dislocation strengthening, Orowan strengthening, and load transfer strengthening.

## 1. Introduction

Because titanium alloys have the advantages of a low density, good mechanical properties, and corrosion resistance, they are widely used in fields such as automobiles, aerospace, biomedical equipment, and military defense [1,2,3,4]. However, the development of these and other industries has led to higher requirements for strength, modulus, wear resistance, and other properties of titanium alloys. To improve these properties, reinforced phases are commonly used to strengthen the titanium alloy matrix to fabricate TMCs [5]. For example, TMCs with good mechanical properties at both room temperature and high temperatures were prepared by using ceramic reinforcements such as SiC [6], TiC [7], and TiB [8,9]. In the above studies, hard reinforcements were added to enhance the strength of the materials, but this led to a decrease in plasticity [10]. Among the known reinforcement materials, graphene has the highest strength and elastic modulus, which are expected to give metal matrix composites good plasticity and strength [11].

Graphene, as a typical two-dimensional nanomaterial, has excellent properties, such as ultra-high strength (130 GPa), high thermal conductivity (3000–5000 W/[m ·k]), and so on [12,13,14]. Therefore, it is considered to be an ideal reinforcement material in different metal [15] matrices, such as those of Mg [16], Al [17], Ti [18], and others. The premise of maximizing the superior performance of graphene is the high load transfer from the metal matrix to graphene, which depends on the uniform dispersion of graphene in the matrix and the interfacial bonding between the two [19,20]. In existing studies, graphene has been uniformly dispersed in metal matrices, such as in flake powder metallurgy [21]. However, poor adhesion between graphene and the matrix remains a key problem [22,23,24]. Therefore, new forming technologies are needed to prepare composites with good matching of strength and plasticity. Chen et al. [25] prepared carbon nanotubes (CNTs)/Al composites by spark plasma sintering (SPS) and hot extrusion while optimizing the interface by changing the sintering temperature. The results showed that higher sintering temperatures led to a closer interface and a higher tensile strength. The traditional processing methods (casting, powder metallurgy) and additive manufacturing (selective laser sintering, laser metal deposition, SLM) prepare TMCs in the process of high temperatures. Titanium and carbon have a certain reaction tendency at a high temperature, and TiC is formed in situ. The results show that TiC can improve the mechanical properties of TMCs, and the chemical bond between TiC and the matrix is a coupled interface or semi-coherent interface. Given the strong proclivity of Ti–carbon chemical reactions [26], and due to its fast melting and quick cooling features, the laser melting method provides an excellent thermal resource for the manufacture of GNPs/Ti composites when compared to alternative material processing methods [27,28,29]. At the same time, there are few studies on strengthening the TC11 titanium alloy by GNPs. In this study, a TC11 titanium alloy and GNPs/TC11 composite was prepared by SLM, and their microstructure and tensile properties at room temperature were studied, which provides a reference for the preparation of graphene TMCs with better properties, as well as a basis for their application.

## 2. Results

### 2.1. Phase Analysis of Powder and Composites

**Figure 1:**
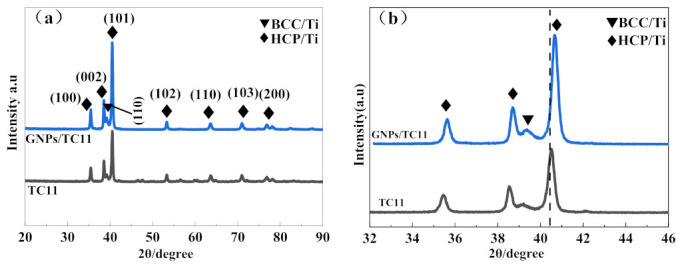
XRD patterns: (**a**) TC11 alloys and titanium matrix composite and (**b**) a partial view of (**a**).

Figure 1 shows the XRD diffraction results of the SLM-formed TC11 titanium alloy and GNPs/TC11 TMCs. It was observed that the XRD diffraction peaks were all composed of HCP/Ti and BCC/Ti. Based on our calculations, the lattice constants a and c of HCP/Ti in the two materials were 0.2913/0.46167 nm and 0.2902/0.46317 nm, respectively, which were close to the theoretical values of the α’ phase. Thus, the main phases were the α’ phase and a small amount of the β phase. The diffraction peaks of the composite material and TC11 were similar, which indicated that the addition of GNPs did not change the main phase composition of the TC11 titanium alloy material, but the diffraction peak of the composite material still weakly moved towards a higher 2θ angle in the local magnification area, as shown in Figure 1b. According to the Bragg equation, the larger the diffraction angle 2θ, the smaller the lattice constant, which indicated that the addition of GNPs caused the α’ phase to undergo lattice distortion, making all lattice constants smaller. In contrast, no carbide diffraction peak was found in the spectrum of the TMCs, which was most likely due to the addition of too little graphene or being limited by the detection accuracy of XRD.

**Figure 2:**
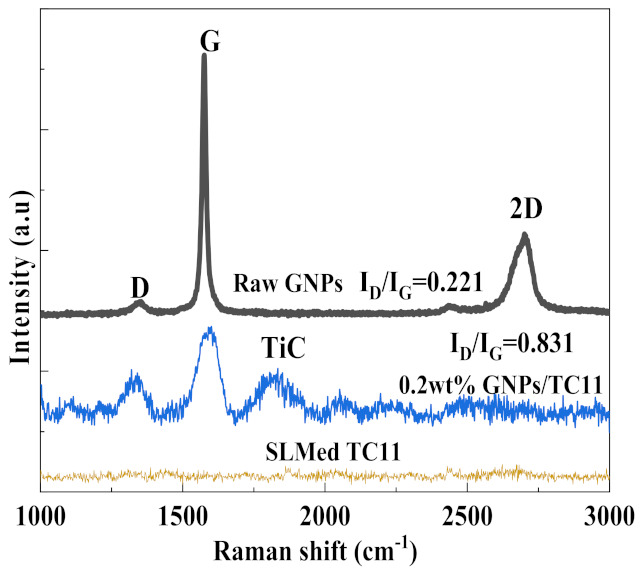
Raman spectra of raw graphene and graphene in the SLM-formed GNPs/TC11 composite.

To further explore whether the GNPs and TC11 produced other substances, and if they did, whether the reaction is complete, the graphene powder and GNPs/TC11 composites were analyzed via Raman spectroscopy, as shown in Figure 2. The phenomenon that there was no characteristic Raman peak in the pure TC11 titanium alloy was caused by the detection principle of Raman spectroscopy. In the composite material, characteristic D and G peaks of graphene appeared, as did TiC peaks. The structural defect density of graphene in the composite material was determined using the ratio of D peak to G intensity (I_D_/I_G_), as seen in Figure 2 [30,31]. This ratio was compared with that of the original graphene; the larger the ratio, the higher the density of structural defects. The ratio of the composite material was 0.812—obviously larger than the ratio of the raw graphene at 0.221—which indicated that the defect density of graphene increased after laser forming. Since SLM forming is a process of rapid cooling and solidification, when the laser beam moves to other areas, the material quickly cools down to below the reaction temperature of Ti and C, thereby preventing the continued generation of TiC. In addition, the added GNPs were multilayer graphene, so not all GNPs will react to form derivatives. Furthermore, some graphene could also maintain its complete structure before laser melting and forming so that it could play a strengthening role in the composite materials.

### 2.2. Microstructure

**Figure 3:**
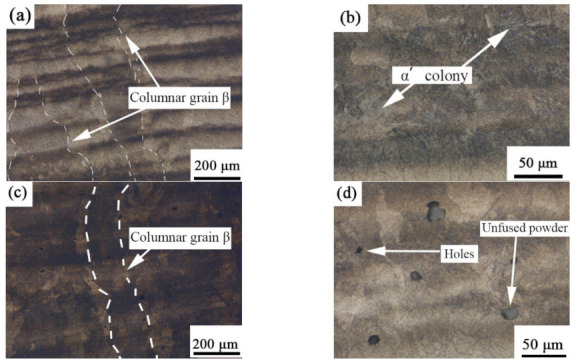
Metallographic diagram of longitudinal section (parallel to deposition direction) of SLM-formed samples: (**a**,**b**) TC11 titanium alloy; (**c**,**d**) GNPs/TC11 TMCs.

Figure 3 shows the metallographic morphology of the SLM-formed TC11 titanium alloy and GNPs/TC11 titanium matrix composite in the longitudinal section (i.e., parallel to the forming direction). It can be seen from the figure that the microstructure of TC11 and GNPs/TC11 composites was relatively uniform and dense, and there were no common defects such as large-area pores, cracks, or non-fusion, which indirectly showed that the process parameters selected in the experiment were reasonable and provided good formability. The metallographic morphology of the longitudinal section can be observed; the β columnar crystal runs through the multi-layer deposition layer, with a width of 80–160 μm and a length of 0.4–1.4 mm. In the process of SLM forming, the temperature of the molten pool varied with the superposition of the deposition layer, and the molten metal solidified upward along the deposition direction. When the laser beam melted a new layer of powder, the top of the columnar crystal formed in the previous layer was affected by the laser so that the bottom of the columnar crystal became a new layer of the directionally solidified nucleus. Therefore, the columnar crystals showed a trend of epitaxial growth. This was consistent with studies by Thijs [32], where the direction of grain growth in the SLM forming process depended on the direction of heat conduction. Figure 3b shows a magnified image of the columnar crystal. Combined with the XRD data, it was determined that the columnar crystal was filled with an acicular martensite α’ phase. This kind of structure was also characteristic of TC4 formed by SLM. Figure 3c shows a metallographic diagram of the TC11/GNPs; the addition of graphene did not change the microstructure of the titanium matrix composite, which was similar to the metallographic morphology of TC11 (Figure 3a). However, as seen in Figure 3d, there were unfused pore defects, which were caused by the agglomeration of graphene [33].

**Figure 4:**
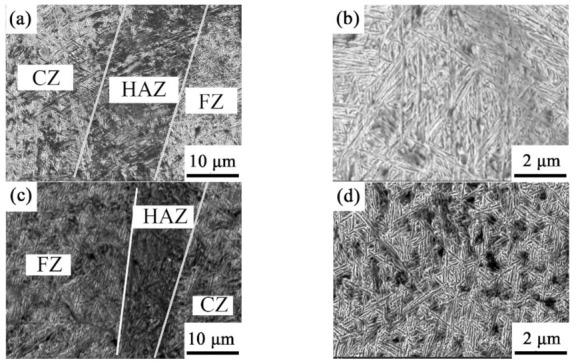
SEM morphology of TMCs: (**a**,**b**) TC11 titanium alloy; (**c**,**d**) GNPs/TC11 TMCs.

In GNPs/TC11 composite specimens, three distinct particle size regions, namely coarse zone (CZ), heat-affected zone (HAZ), and fine zone (FZ), could be observed, as they are in pure TC11 specimens. Figure 4b shows a high-magnification SEM image of CZ in Figure 4a. Combined with the XRD pattern of Figure 1, it can be seen from Figure 4d that the microstructure was mainly composed of acicular martensite α’ phase, and a net-basket structure micromorphology, as well as a small amount of Widmanstätten colony distribution, were mainly present. However, the Widmanstätten colony was reduced compared with the TC11 sample, and the grain size was smaller than that of the TC11 titanium alloy. The acicular martensite became shorter, indicating that grain refinement had occurred. This was consistent with the decreased lattice constants of the GNPs/TC11 composites via XRD.

**Figure 5:**
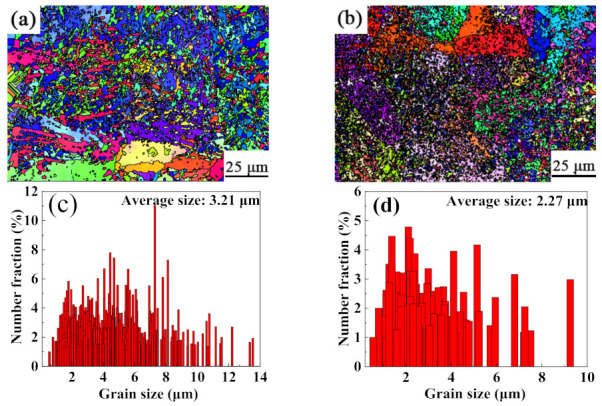
Grain orientation and size distribution of SLM-formed alloys: (**a**,**c**) TC11 titanium alloy; (**b**,**d**) GNPs/TC11 titanium matrix composite.

Figure 5 shows the grain orientation and particle size statistics of the TC11 titanium alloy and GNPs/TC11 composite. It can be seen from Figure 5a that the microstructure of the TC11 titanium alloy was coarse-grained. The orientation diagram was mainly blue and red, and the former occupied the largest proportion and had a strong (11–20) orientation. However, the grain morphology of the GNPs/TC11 composite was elongated and lath-like. The red and green areas slightly increased, while the blue areas were greatly reduced. The (0001) and (10–10) orientations were enhanced, while the (11–20) orientations were weakened. This indicated that the addition of graphene led to changes in grain orientation. According to grain size distribution diagrams (Figure 5b,d), the average grain size of the TC11 samples was 4.34 μm, while that of the GNPs/TC11 composite was 2.27 μm (i.e., the grains were refined). On the one hand, the grain refinement was due to the morphological characteristics of the crystal structure, which mainly depended on two factors, namely, temperature gradient (G) and growth rate (R). The morphology of the microstructure before solidification is described by the ratio of G/R, while the cooling rate of the molten pool is expressed by the value GR, which determines the size of the microstructure. Because graphene has a high thermal conductivity (5000 W·M−1·K−1)—much higher than that of the TC11 titanium alloy (15.24 W·M−1·K−1)—the GR value of the composite material was much higher than that of the TC11 titanium alloy, indicating that the addition of graphene improved the cooling rate of the molten pool. Thus, the grains could not continue to grow, resulting in a decrease in grain size. On the other hand, graphene becomes a heterogeneous nucleation substrate during the forming process, which can greatly increase the number of non-uniform nucleation events. When the number of crystal nuclei reaches a certain level, they can significantly inhibit the growth of grains.

**Figure 6:**
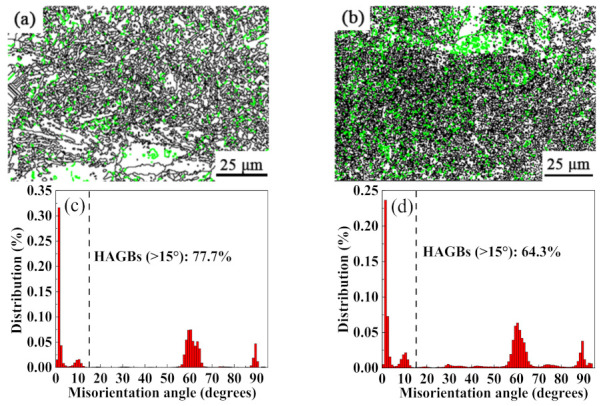
Distribution of grain boundary misorientation and HAGB ratio of SLM-formed alloys: (**a**,**c**) TC11 titanium alloy; (**b**,**d**) GNPs/TC11 titanium matrix composite.

Figure 6 shows the grain boundary orientation distribution of the TC11 titanium alloy and GNPs/TC11 TMCs. The grain boundary angles can be divided into two types: high-angle grain boundary (HAGB, black), with a large difference in grain orientation (>15°), and low angle grain boundary (LAGB, green), with a small difference in grain orientation (>15°).

The grain boundary orientation difference distribution diagram can be used to evaluate dislocation density. Within the framework of the classical dislocation model, the higher the content of LAGBs, the higher the dislocation density [34]. Figure 6b,d show that the content of HAGBs in GNPs/TC11 composites decreases while that of LAGBs increases, indicating a high dislocation density. The increase in dislocation strength was due to the large difference in elastic modulus between the TC11 titanium alloy and graphene, as well as their coefficients of thermal expansion. The large difference in thermal deformation between the two materials resulted in the formation of high-density dislocations at the interface, which stored the deformation energy.

**Figure 7:**
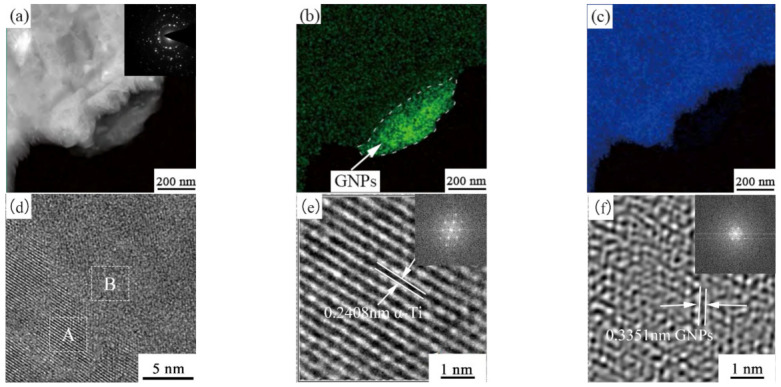
GNPs/TC11 composite interface combined with TEM images: (**a**) TEM diagram of GNPs/TC11 open field interface; (**b**) C element distribution map; (**c**) Ti element distribution map; (**d**) HRTEM diagram of GNPs/TC11 interface; (**e**,**f**) FFT and IFFT diagrams of rectangular frames A and B in (**d**), respectively.

Figure 7a shows a high-angle bright-field diagram of the GNPs/TC11 composite. Two phases of different contrasts were seen, the dark part being the Ti matrix and the translucent light-colored part being graphene; this was verified by the electron diffraction patterns of graphene (illustrated in Figure 7a), and the element distributions of Ti and C in the EDS surface sweeps in Figure 7b,c. Figure 7d show a higher resolution TEM (HRTEM) image of the interface. Figure 7e,f show inverse fast Fourier transformation (IFFT) and fast Fourier transformation (FFT) images of A and B in Figure 7d, respectively. It can be seen that the lattice spacing was 0.2408 nm, which corresponds to the (0110) [35] crystal plane of α-Ti, with a closely arranged hexagonal crystal structure. When the spacing of the crystal planes was 0.3351 nm, the phase was identified as graphene. The lattice spacing was smaller than the theoretical values (α-Ti = 0.2555, GNPs = 0.34), which was caused by the lattice mismatch between the matrix of stone GNPs and Ti (33.4%, much larger than 5%). Therefore, the crystal planes of graphene and Ti were very poor, and the interface may have been mainly composed of metallurgical and mechanical bonding. However, it can be seen from Figure 7a,d that the interface between the GNPs and Ti matrix was tightly bonded, and there were no pores, cracks, or impurity phases. As seen in Figure 7d, part of the α-Ti lattice fringes disappeared gradually at the interface, which made it difficult to distinguish the different phases. This was because the GNPs were embedded in the Ti matrix, which made the lattice fringes of different phases overlap and blur with each other such that there were no obvious boundaries.

**Figure 8:**
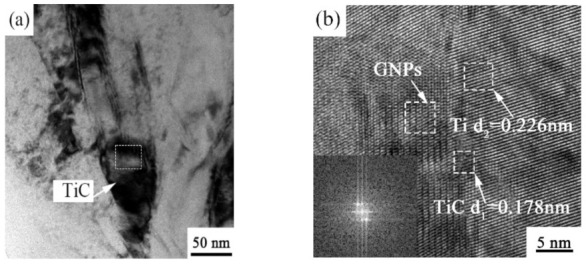
TEM images of GNPs/TC11 composite TiC: (**a**) TEM morphology bright field image of TiC; (**b**) HRTEM image of the enlarged area in (**a**).

With the increase of temperature, the reaction between Ti and graphene intensifies, and the content of TiC in the composite increases. In order to further explore whether there are other derivatives between the interface of Ti matrix and carbon in the composite and to observe the bond between the interface, Figure 8a shows a bright field diagram of the in situ TiC particles dispersed in the Ti matrix, and Figure 8b shows an HRTEM image of the boxed region in Figure 8a. It is shown that the crystal plane spacing of Ti region is 0.228 nm, which corresponds to the (101) crystal plane of Ti. The plane spacing of TiC can be obtained as 0.178 nm in the transition region between GNPs and Ti, and the crystal plane spacing of 0.332 nm near TiC indicated that its phase was graphene; thus, not all graphene reacted with Ti. Among TC11, GNPs, and TiC, the interface bonding was good, and there were no defects. This was consistent with the results obtained by Mu et al. [18] in their study of the interface of multilayer graphene-reinforced Ti matrix composites. Importantly, because graphene was embedded in the grain boundary of the Ti matrix, it inhibited grain growth due to the migration of the grain boundary. However, the grain boundary migration was accomplished through the diffusion of atoms in the grain and across the grain boundary. Graphene embedded in the grain boundary of the Ti matrix acted as a barrier to prevent the diffusion of atoms so that the grain boundary could not move, hindering grain growth. The driving force of grain boundary migration is calculated by the Laplace equation, and the expression is as follows:

(1) $$F=\gamma \left(\frac{1}{\rho_{1}}+\frac{1}{\rho_{2}}\right)$$

where γ is the free energy of the grain boundary per unit area, and $\rho_{1}$ and $\rho_{2}$ are the two principal curvature radii of the grain boundary. Therefore, greater values of $\rho_{1}$ and $\rho_{2}$ mean a smaller force driving the migration of the grain boundary, making it more difficult to move. If the grain boundary is flat, the curvature is 0, the radius of curvature is close to infinity, and the straight grain boundary stagnates. Due to the excellent properties of graphene (e.g., tensile strength = 125 GPa and elastic modulus = 1.1 TPa), the curvature radius of the grain boundary increases, which straightens it. This reduces the driving force of grain boundary migration and restrains grain growth. When the size of graphene is much smaller than the grain boundary length, the graphene does not hinder grain boundary migration as much, and the grain boundary curvature radius remains constant. When the grain boundary crosses the graphene to form a new grain boundary, the change in grain boundary energy is as follows:

(2) ΔG=γA

where A is the area of the graphene. Therefore, when the grain boundary crosses the graphene, the area of former increases, as does the grain boundary energy, so that graphene can produce a certain resistance to the grain boundary and drag it. Thus, the grain boundary migration rate decreases or even becomes 0, which inhibits the growth of grains.

### 2.3. Tensile Properties

**Figure 9:**
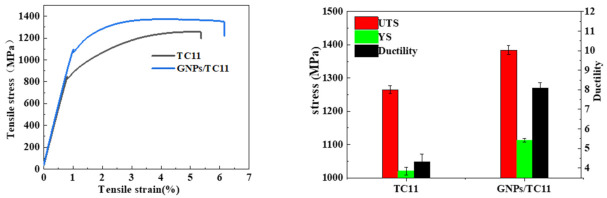
Stress–strain curves and tensile properties diagram of TC11 titanium alloy and GNPs/TC11 composite samples.

Figure 9 shows stress–strain curves and bar charts of the tensile properties of the TC11 titanium alloy and GNPs/TC11 composite samples. It can be seen that the addition of graphene significantly improved the tensile properties of the samples. The tensile strength and yield strength of the TC11 samples were 1265 ± 12 MPa and 1020 ± 15 MPa, respectively, while the tensile strength of the GNPs/TC11 composite specimens increased to 1384 ± 11 MPa—7% higher than that of the TC11 titanium alloy. In addition, it should be noted that the elongation of the GNPs/TC11 composite was also improved from 4.3% to 8.1%, indicating that the GNPs/TC11 composite had excellent mechanical properties.

**Figure 10:**
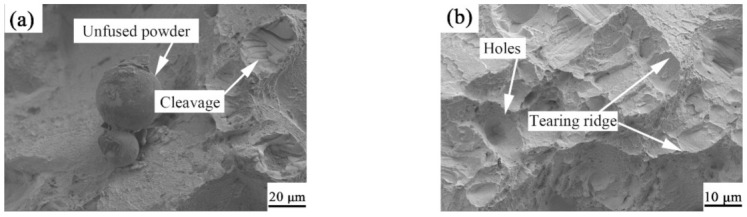

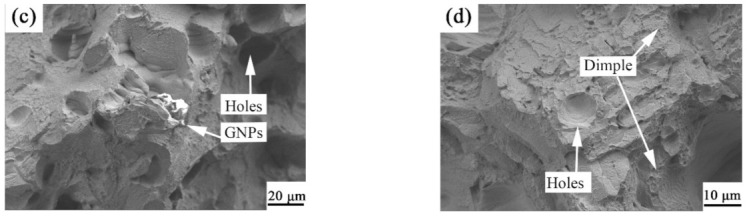
Fracture topography of stretched parts: (**a**,**b**) TC11 titanium alloy; (**c**,**d**) GNPs/TC11 titanium matrix composite.

To better analyze the fracture mechanism of the TC11 titanium alloy and GNPs/TC11 composites from their microstructure morphology, the fracture morphology of the tensile parts was observed by SEM. As shown in Figure 10a, unfused spherical TC11 powder, quasi-cleavage fracture characteristics, and the existence of dimples were observed at low magnification, indicating a mixed fracture mode of plasticity and brittleness. As seen in Figure 10c, the tear edge size became smaller, the dimple size became larger, and bright and dark materials were observed. After EDS point scanning, all the C elements were known to be GNPs; thus, it was determined that the fracture mode of the GNPs/TC11 composites was still a brittle-plastic mixed fracture. Furthermore, there was pulled-out and torn graphene at the interface, which indicated that another reason for the improvement of the tensile properties of the composites was the strengthening mechanism of the load transfer.

## 3. Discussion

At present, domestic and foreign researchers believe that the strengthening mechanism of graphene-reinforced TMCs may result from the following: (1) additional dislocation density generated in the matrix due to the large difference in the thermal expansion mismatch between the GNPs and Ti matrix and their derivative TiC and Ti matrices, resulting in the strengthening effect; (2) a strengthening mechanism of the load transfer from the matrix to the GNPs or TiC particles in the nano-GNPS reinforced metal composites; (3) the occurrence of fine grain strengthening with a smaller grain size; or (4) Orowan dislocation ring strengthening induced by dispersed nanoparticles of TiC and GNPs in the matrix. In this study, the GNPs/TC11 composite was used to analyze the strengthening mechanism.

### 3.1. Dislocation Strengthening (CET)

In general, the greater the dislocation density in the matrix, the more likely it is for mutual delivery in the process of dislocation movement to occur, which forms cut steps that result in an entangled dislocation together to form a dislocation wall, thus hindering the dislocation movement. Thus, the continuous plastic deformation of the matrix is blocked, and finally, the strength of the material is improved. In this study, due to the characteristics of thermal stress caused by rapid solidification in the SLM forming process, as well as the mismatch of the thermal expansion coefficient and elastic modulus between GNPs (0.9 × 10^−6^ K^−1^) and the Ti matrix (8.8 × 10^−6^ K^−1^) during forming, the dislocation density within the Ti matrix increased. At the same time, the difference of thermal expansion coefficient between the Ti matrix and GNPs caused multidirectional stress at their interface, leading to the increase in dislocation density. This is also called thermal mismatch reinforcement. The increase in intensity caused by the CET mechanism is expressed by the following formula [16,36]:

(3) $$\Delta \sigma_{CTE}=\sqrt{3}\beta Gb\sqrt{\rho }$$

(4) $$\rho =\frac{D\times V_{GNPs\;}\times \left(\Delta C\times \;\Delta T\right)}{b\left(1-V_{GNPs}\right)\times t}$$

where $\Delta \sigma_{CTE}$ is the strength increment generated by dislocation strengthening (MPa), β is the dislocation strengthening coefficient, G is the shear modulus of the Ti matrix (4.3 × 10^4^ MPa), *b* is the anchor vector of the Ti matrix (0.289 nm), ρ is the dislocation density, and D is relative to the flake strengthening phase, whose value is generally eight.

### 3.2. Load Transfer Strengthening

After the composite is stretched, the Ti matrix, which bears the tensile load, transfers it to the reinforced phase via shear stress along the interface between the reinforced phase and the matrix. The shear force on the interface parallel to the load direction is balanced by the normal force on the intersecting surface perpendicular to the load direction. When the shear force is greater than the force that the interface can bear, GNPs transfer the load to the matrix for limited extension. If the shear force is less than the normal force that the GNPs can bear, interfacial debonding occurs because the strength of the GNPs (GPa) is much higher than that of the Ti alloy matrix (MPa), and the fractured GNPs typically interfacially debond in the tensile section. Considering the mechanical strength of the GNPs at 130 GPa, the shear force can hardly exceed the normal force that the GNPs can bear; thus, the combination of interfaces is very important for load transfer. Because researchers study the strength increment of load transfer strengthening produced by GNPs in composites according to the actual situation, the strengthening formulas will be different, but the yield strength increment of composites strengthened by the load transfer strengthening mechanism can be calculated by a modified shear lag model. The calculation expression is as follows [37]:

(5) $$\Delta \sigma_{LT}=0.5V_{GNPs}\sigma_{ym}$$

where $\sigma_{ym}$ is the yield strength of the Ti matrix, and $V_{GNPs}$ is the volume fraction of GNPs. Because the yield strength of the Ti matrix was 1009 MPa, when the content of GNPs was 0.2%, the yield strength of the composite increased by 11 MPa via the load transfer strengthening mechanism.

### 3.3. Fine Grain Strengthening

The fine grain strengthening mechanism in the strengthening mechanism of composites is generally considered to be an important method. According to the statistical results of the particle sizes obtained by an electron backscattered diffraction (EBSD) test, as seen in Figure 7, the average grain size of the 0.2 wt% GNPs composite was 2.27 µm, which was smaller than that of the TC11 material formed by SLM. The cause of grain refinement has been explored previously and will not be analyzed here. The Hall–Petch formula is the theoretical model used to calculate the grain refinement effect; the formula is as follows [18,38]:

(6) $$\Delta \sigma_{HP}=k_{hp}\left(d_{c}^{-0.5}-d_{m}^{-0.5}\right)$$

where $k_{hp}$ is the Hall–Petch coefficient of Ti (0.68 MPa·m^1/2^ [39]), and $d_{c}$ and $d_{m}$ are the average grain sizes of the GNPs/TC11 composite and TC11, respectively. According to this calculation, the grain refinement increased the yield strength increment by 0.41 MPa·m^1/2^.

### 3.4. Orowan Looping Strengthening

From SEM images of the GNPs and in situ nano-TiC particles observed in the tensile section, as seen in Figure 8 and Figure 10, respectively, it can be seen that there were second-phase particles of the above two substances in the composites. During the deformation of the material, the dislocation lines could not directly pass through the two phases, and the dislocation lines bent under the action of external forces, forming a dislocation ring centered on the GNPs and TiC. The effect led to an increase in lattice distortion for the dislocation influence zone and restrained the movement of dislocations. As a result, the strength of the composite was improved. This kind of strengthening is called the Orowan strengthening effect, and its strengthening contribution can be calculated by the Orowan–Ashby equation [16]:

(7) $$\Delta \sigma_{Orowan}=\frac{0.13G_{m}b}{\lambda }\ln\frac{d}{2b}$$

where *λ* and *d* are the average spacing and particle diameter of the second-phase particles (nm), respectively, *b* is Poisson’s modulus of the Ti matrix (0.34 nm), and $G_{m}$ is the shear modulus of the Ti matrix (54.1 GPa). According to the above formula, the smaller the size and spacing of the second-phase particles, the more obvious the strengthening effect. The yield strength, as a result of this strengthening mechanism, was 13.2 MPa.

(8) $$\sigma_{YS}=\sigma_{1}+\;\Delta \sigma_{CET}+\;\Delta \sigma_{LT}+\;\Delta \sigma_{HP}+\;\Delta \sigma_{Orowan}$$

where$\;\sigma_{1}$ is the yield strength of the TC11 titanium alloy. The yield strength of the GNPs/TC11 composite calculated by this formula was close to that obtained by the tensile test.

## 4. Materials and Methods

**Figure 11:**
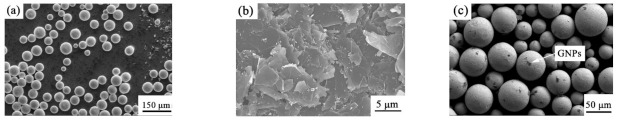
SEM morphological images: (**a**) TC11 powder; (**b**) GNPs; and (**c**) GNPs/TC11 composite powders.

TC11 spherical powder with an average particle size of 46.13 μm was used as the starting material; its chemical composition is shown in Table 1. In Figure 11a,b, scanning electron microscope (SEM) images of the titanium powder are shown, with spherical-shaped particles and graphene, respectively. As can be seen from Figure 1b, the thin graphene sheets overlapped with each other, and they had a large specific surface area, which was measured to be approximately 375.1 m^2^/kg using a particle size analyzer.

The GNPs/TC11 composite powders were prepared via powder mixing, stirring, drying, and other processes. Since graphene and the Ti matrix had a large interface contact area, it was more challenging to uniformly disperse the former into the latter compared to other reinforcing materials [39]. Therefore, low mass-fraction GNPs (0.2 wt%) were selected in this study. First, the TC11 powder (2 kg) and GNPs (4 g) were mixed before adding absolute ethanol (200 mL) and stirring to form a slurry. This was placed into a homogenizer operated at speeds of 500, 1100, and 1700 rpm. The mixed slurry was stirred for 90 s and finally placed into a dryer at 80 °C. Figure 1 shows the morphology of the mixed powder; compared to the TC11 powder, black flakes were distributed on its surface. An energy dispersive spectrometer (EDS) energy spectrum analysis of the black flakes showed the presence of carbon due to the addition of the GNPs, which verified that the GNPs were evenly distributed on the surface of the TC11 particles.

In this study, the metal 3D printing machine BLT-S200 developed by Xi’an Bright Corporation was used to prepare the TC11 and GNPs/TC11 composite material samples. The SLM parameters were optimized and set as follows: the laser power was 280 W, the laser scanning speed was 1200 mm/s, the hatch spacing was 0.1 mm, the layer thickness was 30 μm, and the checkerboard scanning strategy was used. The composite material sample was an 8 mm × 8 mm × 8 mm cubic test block, and the tensile samples were transformed into a 50-mm-long, 2.5-mm-thick dog bone-shaped specimen with gauge length of 20 mm. The direction of the long side of the tensile specimen was parallel to the plane of the substrate.

The phase of the polished cross-section perpendicular to the forming direction during SLM was analyzed by a Rigaku D/max-BB X-ray diffractometer (XRD) within a scanning range of 20~90°. After etching the sample with the Keller reagent (101 mL H_2_O + 5 mL HNO_3_ + 2 mL HF) for 15 s, its microstructure and fracture morphology were analyzed by a Leica DM2700M metallographic microscope (OM) and a JSM-IT500LA SEM. The distribution of GNPs in the Ti matrix was observed by a JEM-F200 transmission electron microscope under 200 kV, and the microstructures of dislocations, derivative compounds, and the interface were studied. The orientation and size of the crystal were analyzed by HK Channel 5 software. The stretched parts were stretched at room temperature at a tensile rate of 1 mm/min using a W9W-50 universal testing machine until breaking.

## 5. Conclusions

In this study, the microstructure and mechanical properties of TC11 titanium alloy and GNPs/TC11 composites formed by SLM were investigated. The optimal process parameters were a laser power of 280 W, scanning speed of 1200 mm/s, scanning distance of 0.1 mm, and layer thickness of 0.03 mm. The microstructure of the TC11 titanium alloy and GNPs/TC11 composites formed by SLM were of the acicular martensite α’ phase, and the size of which in the graphene-controlled composites was finer than that of the TC11 titanium alloy. In addition, the proportion of low-angle grain boundaries in the composites was larger than that of the TC11 titanium alloy. Ti and GNPs formed TiC in the composites-forming process. Both GNP and TiC enhance the mechanical properties of GNPs/TC11 composites. The strength improvement of the GNPs/TC11 composite was mainly due to a joint effect of dislocation strengthening, load transfer strengthening, fine grain strengthening, and Orowan strengthening mechanisms, but the effect of the fine grain strengthening on the strength of the GNPs/TC11 composite was negligible.

## Figures and Tables

**Table 1:** Chemical composition of TC11 titanium alloy powder (wt%).

Element	Ti	Al	Mo	Zr	Si	Fe	C	H	O
Mass fraction %	Bal	6.5	3.56	1.72	0.277	0.008	0.07	0.003	0.12

## Data Availability

Authors can confirm that all relevant data are included in the article.
